# Artificial intelligence identifies worsening heart failure by voice analysis: a pilot study

**DOI:** 10.1093/geroni/igaf122.666

**Published:** 2025-12-31

**Authors:** Yang Yi, Bochao Zhao, Dire Ying, Keyao Liu, Wendong Xiao, Qiaoqin Wan

**Affiliations:** Peking University, Beijing, Beijing, China (People’s Republic); University of Science and Technology Beijing, Beijing, Beijing, China (People’s Republic); Peking University, Beijing, Beijing, China (People’s Republic); Peking University, Beijing, Beijing, China (People’s Republic); University of Science and Technology Beijing, Beijing, Beijing, China (People’s Republic); Peking University, Beijing, Beijing, China (People’s Republic)

## Abstract

Effective out-of-hospital monitoring is essential for reducing heart failure (HF) readmission rates. Emerging evidence suggests that voice biomarkers may serve as non-invasive indicators for HF monitoring. The objectives of this study were to (1) identify HF-specific speech tasks and acoustic features; and (2) validate the reliability of mobile phone recordings in HF monitoring. Recordings were collected in three phases from 160 HF patients. Phase I focused on simplifying speech tasks based on feasibility and correlation with NT-proBNP levels, with recordings taken on the same day as NT-proBNP testing. Phase II aimed to assess the cross-device stability of acoustic features through simultaneous recordings using both professional equipment and smartphones. Phase III sought to develop machine learning models to differentiate patient statuses at admission, mid-hospitalization, and discharge, and identify speech tasks and acoustic features most relevant to HF. Out of 11 tasks, 4 were retained for the final model. The accuracy, sensitivity, specificity, and area under the curve for distinguishing admission and discharge were 0.82, 0.85, 0.78, and 0.85, respectively; for mid-hospitalization and discharge, these were 0.82, 0.79, 0.85, and 0.88. Key features included Mel-frequency cepstral coefficients, chroma, fundamental frequency, frequency-domain, energy, and glottal features. No significant acoustic differences were observed between mobile phone brands. This study established a feasible, reliable set of speech tasks, identified HF-specific acoustic features, and validated mobile phone recordings for HF monitoring, supporting the potential for remote, at-home HF patient monitoring.

